# Atorvastatin attenuates experimental contrast-induced acute kidney injury: a role for TLR4/MyD88 signaling pathway

**DOI:** 10.1080/0886022X.2017.1361838

**Published:** 2017-08-14

**Authors:** Rongzheng Yue, Chuan Zuo, Jing Zeng, Baihai Su, Ye Tao, Songmin Huang, Rui Zeng

**Affiliations:** a Department of Nephrology, West China Hospital, School of Clinic Medicine, Sichuan University, Chengdu, PR China;; b Department of Rheumatology and Immunology, West China Hospital, School of Clinic Medicine, Sichuan University, Chengdu, PR China;; c Department of Internal Medicine, West China Hospital, School of Clinic Medicine, Sichuan University, Chengdu, PR China;; d Department of Cardiovascular Diseases, West China Hospital, School of Clinic Medicine, Sichuan University, Chengdu, PR China

**Keywords:** Contrast-induced acute kidney injury, atorvastatin, renal failure, TLR4/MyD88, signaling pathway

## Abstract

**Objectives:** To investigate the protective effect of different atorvastatin doses on contrast-induced acute kidney injury and the related mechanism.

**Methods:** Healthy male Sprague–Dawley (SD) rats were randomly divided into the blank control group, experimental control group and different-dose atorvastatin groups. A rat model of contrast-induced acute kidney injury was established. We detected changes in serum creatinine (Scr) and blood urea nitrogen (BUN) before and after model establishment, observed and scored renal tubular injury, analyzed rat renal cell apoptosis, and measure the expression of signal pathway proteins and downstream inflammatory factors.

**Results:** After contrast agent injection, the Scr and BUN levels of the experimental control group were significantly increased, the different doses applied in the atorvastatin group significantly reduced the Scr and BUN levels (*p* < .05) and ameliorated the contrast-induced acute kidney injury (*p* < .05) and significantly reduced Toll-like receptor 4 (TLR4), Myeloid differentiation factor 88 (Myd88), and Nuclear factor kappa-light-chain-enhancer of activated B cells (NF-κB) protein expression and relative mRNA expression levels (*p* < .05) and significantly decreased expression levels of downstream inflammatory factors (*p* < .05).

**Conclusion:** Different atorvastatin doses have protective effects on contrast-induced acute renal tubular injury in rats, possibly by targeting TLR4, suppressing TLR4 expression, regulating the TLR4/Myd88 signaling pathway, and inhibiting the expression of downstream inflammatory factors.

## Introduction

Contrast-induced nephropathy (CIN) is an important complication that occurs during the application of iodinated contrast agent. CIN is the third most common cause of hospital-acquired acute kidney injury after inadequate renal perfusion and nephrotoxic drugs and accounts for 11% of all hospital-acquired renal failure [1]. CIN is not only unfavorable for the patient prognosis but also increases patient medical costs and thus has become an important challenge for clinicians. Because the pathogenesis of CIN remains unclear, no specific and effective prevention and treatment measures are available. Therefore, investigating the molecular pathogenesis of CIN and seeking prevention and treatment measures is an urgent task.

To identify treatment strategies that can prevent the occurrence of CIN as early as possible and suppress the progression of CIN in a timely manner, clinical practices can apply certain preventive measures with confirmed effects, such as the use of isotonic and/or low osmolar contrast media, strengthening hydration, alkalinization of urine, and discontinuing the use of nephrotoxic drugs 24–48 h before surgery. Whether any drugs are effective at preventing CIN is a focus of exploration. Current research has focused on drugs such as N-acetylcysteine (NAC) [2], antioxidants (ascorbic acid) [3], prostaglandin E_1_ [4], adenosine receptor inhibitors (theophylline) [5], low-dose dopamine [6], and calcium antagonists [7]. However, there is no clear evidence to support the therapeutic effect and the effective doses of these drugs. Over the past decade, a number of clinical studies have shown that the protective effect of statins on the kidneys is independent of the lipid reducing effect. Studies on the effects of statins in CIN prevention are often clinical studies containing various bias factors that are difficult to control; thus, the preventive effect of statins against CIN remains undetermined [8–14]. Additionally, because clinical studies investigating the effects of statins on the prevention of contrast-induced acute kidney injury (CI-AKI) occurrence often use large-dose strengthened statin applications, there is a lack of a uniform standard for specific statin doses [15]. Moreover, basic research on the mechanism by which statins effectively reduce CIN is also limited. This study aimed to successfully establish a rat model of CI-AKI, use different atorvastatin doses as an intervention for rat renal tubular epithelial cells treated with a contrast agent, observe the protective effect of atorvastatin on renal injury, and explore the possible molecular mechanisms of CIN occurrence.

## Materials and methods

### Ethical statement

All animal procedures were conducted with prior institutional ethical approval under the requirements of the Chinese Prevention of Cruelty to Animals Act and the Code of Practice for the Care and Use of Animals for Scientific Purposes. Prior clearance was obtained from the Animal Experimentation Ethics Committees of West China Medical Centre and Institutes of Animal Science. The animals of this study were inspected by members of the West China Medical Centre Animal Ethics Committee.

### Animals and materials

Eighty healthy Sprague–Dawley (SD) rats were purchased from the Experimental Animal Center at the West China Medical Center of Sichuan University. The rats were clean grade, 8–10-weeks of age, weighed 250–300 g, and were raised in a constant temperature environment with standardized feeding and unlimited access to drinking water. Atorvastatin was purchased from Pfizer (China) Research & Development Center (Pfizer Shanghai, P.R.China) and suspended into a 4 mg/ml solution with 0.9% saline. Indomethacin was purchased from Tianjin Biochemical Pharmaceutical Co. and suspended into a 2 mg/ml solution with 0.9% saline for use at a dosage of 1 mg/100 g. N-nitro-l-arginine-methyl-ester was purchased from Tianjin Biochemical Pharmaceutical Co. and suspended into a 2 mg/ml solution using phosphate-buffered saline (PBS) for use at a dosage of 1 mg/100 g. Diatrizoate meglumine was purchased from Shanghai Xudong Haipu Pharmaceutical Co.; this reagent is a type of iodinated contrast medium with a 76% iodine content of 370 mg/ml that is used at a dosage of 10 mL/kg.

### SD rats grouping

The 80 SD rats were randomly divided into five groups as follows: blank control group (gastric gavage using normal saline and left femoral vein injection of normal saline) (*n* = 16); experimental control group (gastric gavage using normal saline and left femoral vein injection of indomethacin, N-nitro-l-arginine-methyl-ester and diatrizoate meglumine) (*n* = 16); low-dose atorvastatin group (gastric gavage of 1.5625 mg/kg·d of atorvastatin and left femoral vein injection of drugs as conducted for the experimental control group) (*n* = 16); medium-dose atorvastatin group (gastric gavage of 3.125 mg/kg·d of atorvastatin and left femoral vein injection of drugs as conducted for the experimental control group) (*n* = 16); and high-dose atorvastatin group (gastric gavage of 6.25 mg/kg·d of atorvastatin and left femoral vein injection of drugs as conducted for the experimental control group) (*n* = 16).

### Establishment of a rat model of contrast-induced acute kidney injury

A total of 80 healthy male SD rats were raised under a standardized protocol in a constant temperature environment with unlimited access to water. Before establishing the model, all rats were deprived of water for 12 h but provided free access to food. On the day of modeling, the rats were weighed and anesthetized via intraperitoneal injection of 10% chloral hydrate at a 0.32 mL/100 g dose. The rats were placed in the supine position, and the skin was prepared for the surgery. After disinfection with alcohol, the left femoral vein was dissected, a catheter was inserted and fixed, normal saline were injected every 15 min in blank control group, and indomethacin, N-nitro-l-arginine-methyl-ester and diatrizoate meglumine were injected every 15 min (with diatrizoate meglumine injection time of no less than 5 min at a time) in experimental control group and the other three atorvastatin interventional groups. At 24–48 h after contrast medium injection, rat tail vein blood was collected to measure the serum creatinine (Scr) levels. An increase in the Scr level compared to the pre-injection level greater than 25% of the control value indicated successful modeling.

### Pharmaceutical intervention methods

The experimental groups with different atorvastatin doses received four interventions of atorvastatin by gastric gavage (7 d before modeling, 3 d before modeling, on the day of modeling and 24 h after modeling) with dosages of 1.5625 mg/kg·d, 3.125 mg/kg·d and 6.25 mg/kg·d in the high, medium and low dose groups, respectively. The other two groups received a gastric gavage containing 1 mL of normal saline. The rats in the various groups did not receive hydration treatment. On the day of modeling, the intravenous administration of diatrizoate meglumine was immediately conducted after drug administration for modeling.

### Sample processing and detection indices

Serum sample collection and biochemical parameter measurement: At 0, 12, 24, and 48 h after contrast injection, rat tail vein blood was collected and centrifuged. The top serum layer was collected to measure Scr and blood urea nitrogen (BUN). Kidney tissue sample collection and morphological examination: The kidney was removed from the animal immediately at 48 h after injection of the contrast agent, washed, dried using filter paper, fixed in 10% formalin, made into paraffin sections, and stained with hematoxylin and eosin (HE) to reveal renal tubular structure changes. Double-blind observation was conducted under an optical microscope. For each slice, 10 fields of renal interstitium at a 400X magnification were randomly selected. The renal tubular epithelial cell injury conditions were observed, including the renal tubular lumen, cast, and tubular epithelial cell morphology, and the renal tubular injury severity was scored. The specific scoring criteria [16] were as follows: 0: Normal renal tubules without damage; 1: Mild renal tubular damage (damaged renal tubules <25%); 2: Moderate renal tubular damage (damaged renal tubules 25–75%); 3: Severe renal tubular damage (damaged renal tubules >75%). The mean score of the 10 fields in each section was used as the renal tubular damage score.

### Renal cell apoptosis ratio by terminal deoxynucleotidyl transferase dUTP nick end labeling staining (TUNEL)

Renal cell apoptosis was monitored by TUNEL staining. It was performed with fluorescein UTP according to manufacturer instructions (In Situ Cell Death Detection Kit, Roche Diagnostics, Shanghai, China) for apoptotic cell nuclei and 49 6-diamidino-2-henylindole (DAPI) (Sigma-Aldrich, Shanghai, China) stained all cell nuclei. Apoptotic index (AI) was calculated as the number of TUNEL-positive myocytes/total number of myocytes stained with DAPI from ten fields per renal tissue. All of these assays were performed in a blinded manner.

### Western-blot analysis for TLR4, Myd88, and NF-κB signaling pathway protein expression

Cytoplasmic protein extracts were prepared according to the instructions of the cytoplasmic protein extraction kit. The extracted proteins (35 μg) were subjected to 10% SDS-PAGE. After electrophoresis, proteins were transferred onto PVDF membranes (Millipore, Billerica, MA), and the membranes were then placed into blocking buffer containing nonfat dry milk. Goat monoclonal anti-human TLR4, Myd88, and NF-κB IgG antibodies (final dilution 1:500) were used as the primary antibody, and HRP-conjugated rabbit anti-goat IgG (final dilution 1:10,000) was used as the secondary reagent. An internal control, β-actin, and a protein marker were also used. Detection was performed using an ECL chemiluminescence kit. The data were scanned and analyzed by the Gel Doc 1000 gel imaging system (Bio-Rad Company, Hercules, CA).

### RT-PCR for TLR4, MyD88, and NF-κB mRNA expression

Total RNA was isolated using Trizol reagent (MRC, Cincinnati, OH) according to the manufacturer's instructions. Total RNA (5 µL) was converted to complementary DNA (cDNA) using the Revert Aid™ First Strand cDNA Synthesis Kit. A 5 µL aliquot of the resulting cDNA was used as a template for PCR amplification with the following primers: TLR4, (forward, 5′-TCGGTGGTCAGTGTGCTTGTGG-3′), (reverse, 5′-AAAGCTGAAAGCGGGGCACTCC-3′); Myd88, (forward,5′- TCAACAAGCGAGCGCACCGT-3′), (reverse, 5′-TGAGCGCGACCAACGGTAGA-3′); NF-κB (forward, 5′-CGGAATGTG CAG ATG CAT -3′), (reverse, 5′-ACC CCC ACT ACT CTT GCG GCA-3′); β-actin,(forward, 5′-TCAGGTCATCACTATCGGCAA T -3′), (reverse, 5′-AAA GAA AGG GTG TAA AAC GCA-3′). The amplifications were performed by an initial denaturation (94 °C for 2 min), followed by 45 cycles of denaturation, annealing and extension (94 °C for 20 s, 54 °C for 20 s, 72 °C for 30 s), and a final extension (72 °C for 5 min). β-Actin transcript was also amplified by RT-PCR from the same cDNA template and was used as an internal control. All the primers were designed and synthesized by Genepharma (Shanghai, China). The identity of each PCR product was confirmed by DNA sequencing. The pictures were scanned and analyzed by the Gel Doc 1000 gel imaging system.

### ELISA assays for expression of interleukin -1β (IL -1β), interleukin -6 (IL -6), and monocyte chemoattractant protein (MCP)-1

Renal cortex tissue concentration of IL -1β, IL -6 and MCP-1 were measured using an ELISA and rat specific ELISA kits (Excell, Shanghai, China) according to the kit instructions. A coating plate was then placed in the ELISA reader to read the absorbance of each sample at 450 nm. All optical density values were converted to the final concentration based on mg protein of each samples and expressed as picograms per milligram of total protein.

### Statistical analysis

We used the SPSS 19.0 software for data processing. The results were expressed as the mean ± standard deviation (*X*¯±S). The main statistical indicators all underwent normality and homogeneity of variance tests. Comparisons between two groups were conducted using single factor analysis of variance (ANOVA). A pairwise comparison of multiple sample means with homogeneity of variance was conducted using the Student–Newman–Keuls (SNK) method, and a pairwise comparison of multiple sample means with heterogeneity of variance was conducted using the Games–Howell method. *p* < .05 was considered statistically significant. Raw real-time quantitative PCR data (Ct values) were converted into the relative gene expression levels (2^−ΔΔCt^). A randomization test was used for the pairwise comparisons of various time points between the control group and the experimental groups. The above operations were conducted using the REST (Relative Expression Software Tool) software [17,18].

## Results

### Effect of different atorvastatin doses on the renal functions of rats with contrast-induced acute kidney injury

**Figure 1.**
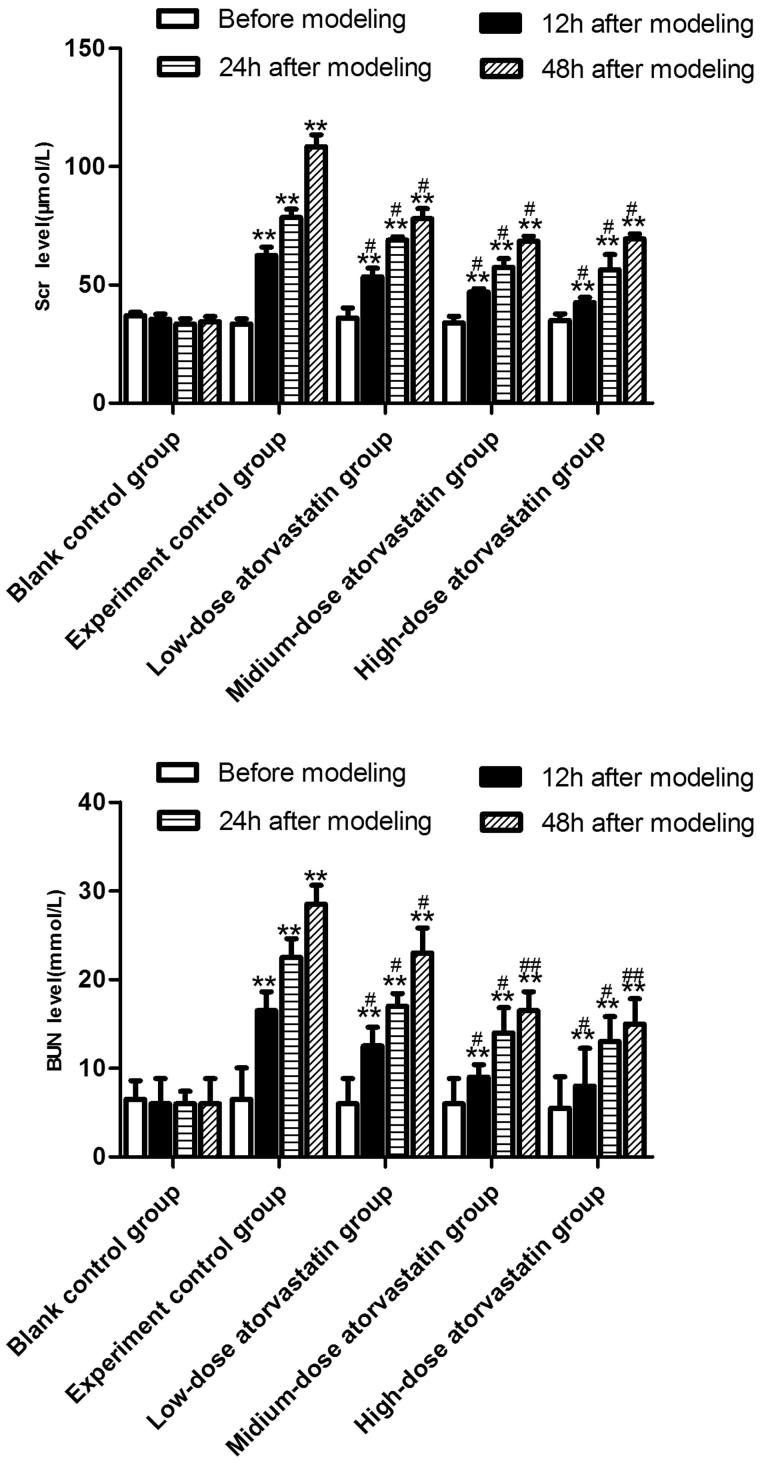
Comparison of changes in the Scr or BUN level 0, 12, 24, and 48 h after contrast agent injection. **Src or BUN level was significantly difference compared to the blank control group (*p* < .01). # or ## Src or BUN level was significantly reduced compared to the experimental control group (#*p* < .05; ##*p* < .01).

The specific results are shown in Figure 1. The Scr and BUN levels of the blank control group did not significantly change with the extension of the modeling time (*p* > .05), whereas the Scr and BUN levels of the experimental control group significantly increased with the extension of modeling time (*p* < .05). Under the effect of atorvastatin, the different doses improved the contrast agent-induced acute kidney injury and significantly decreased the Scr and BUN levels (*p* < .05), but no significant difference was detected between the effects of the medium-dose and high-dose groups on the Scr and BUN levels (*p* > .05).

### Morphological examination of renal tissues

**Figure 2.**
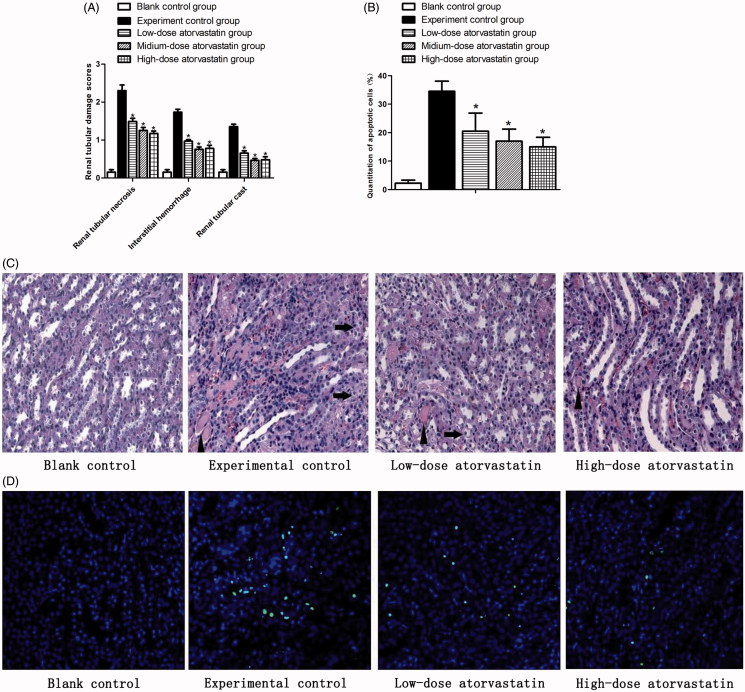
Renal protective effect of atorvastatin on medullary damage and apoptosis in CIN kidney. (A) The histopathologic scores of medullary damage (*p < .05 vs. experimental control group). (B) Quantification of TUNEL-positive nuclei (*p < .05 vs. experimental control group). (C) Representative images of H&E staining under ×400 magnification in the outer medulla. Arrows showed examples of protein casts and tubular vacuolar degeneration/necrosis. Erythrocytes and infiltration of polymorphonuclear cells could be easily observed in the interstitium. (D) Representative images of TUNEL assay under ×400 magnification in the outer medulla.

HE results showed (Figure 2(A, C)) that the renal tissues of the experimental group had high levels of infarction, pathological cast, epithelial cell swelling, transparent, loss, hemorrhage, and neutrophil infiltration after the action of the contrast agent compared with the blank control group (*p* < .05). The different atorvastatin doses improved the outcomes of contrast-induced acute kidney injury and significantly reduced the above-mentioned pathological processes compared with the experiment control group (*p* < .05). However, no significant difference was observed between the effects of the medium-dose and high-dose groups on the above-mentioned pathological processes (*p* > .05).

### In situ TUNEL assay to detect rat renal tissue apoptosis after contrast agent-induced injury

As also shown in Figure 2(B, D), the experimental control group showed a large percent of apoptotic cells 48 h after contrast agent injection (*p* < .05) that almost covered the entire outer medulla area. The different atorvastatin doses all improved the contrast-induced acute kidney injury and significantly reduced the percent of apoptotic cells (*p* < .05), compared with the experiment control group. However, no significant difference (*p* > .05) was also observed between the effects of the medium-dose and high-dose groups on the apoptotic cells.

### Western blotting detection of the TLR4, MyD88, and NF-κB signaling pathway protein expression levels

**Figure 3.**
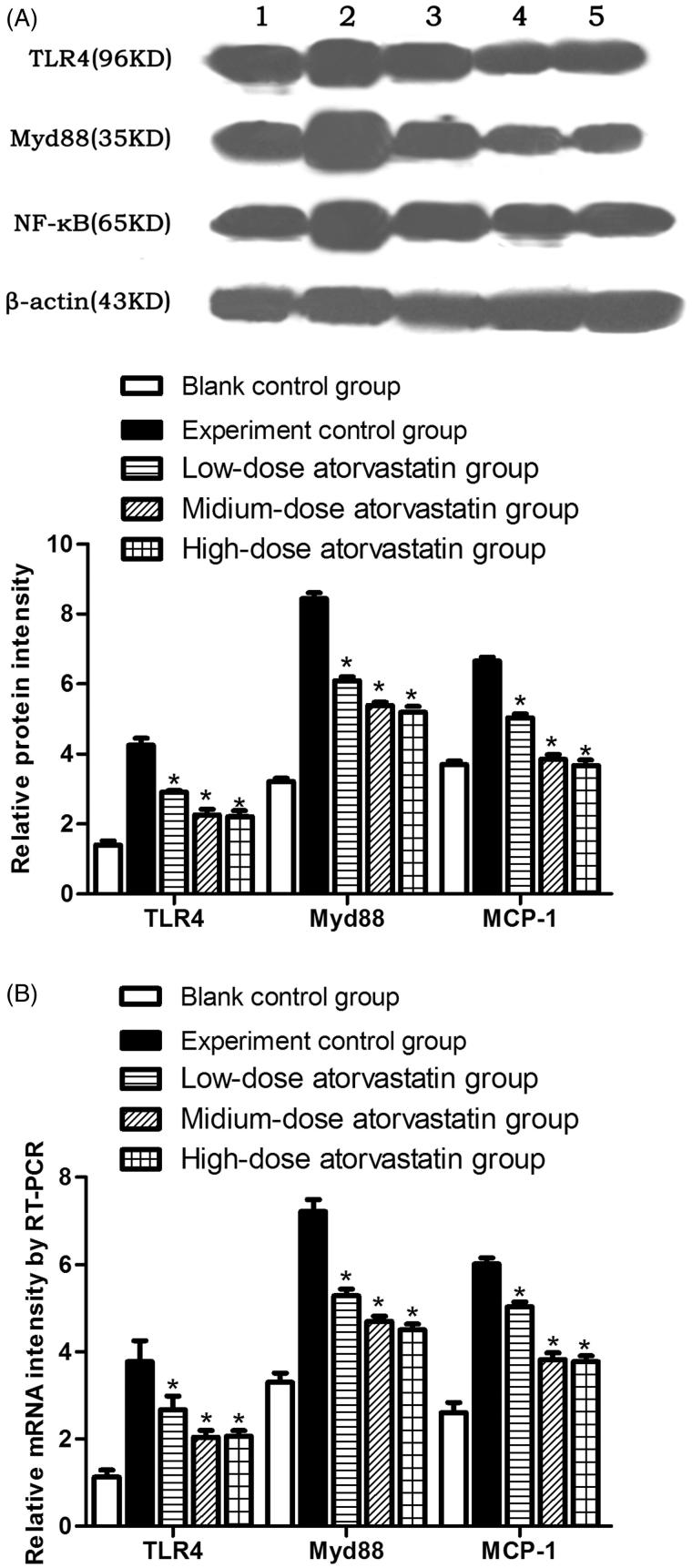
(A) Relative TLR4, Myd88, and NF-κB signaling pathway protein expression levels in the different experimental groups. (B) Relative TLR4, Myd88, and NF-κB mRNA expression levels in the different experimental groups * indicates *p* < .05 compared to the experimental group. All variables were evaluated 48 h after the induction of AKI. (1: Blank control group; 2: Experiment control group; 3: Low-dose atorvastatin group; 4: Medium-dose atorvastatin group; 5: High-dose atorvastatin group.).

At 48 h after contrast agent injection, the experimental control group showed significantly increased TLR4, Myd88, and NF-κB protein expression levels. Atorvastatin significantly reduced TLR4, Myd88, and NF-κB expression. The differences were significant compared with the experiment control group (*p* < .05). However, no significant differences were observed in the protein expression levels between the medium-dose and high-dose groups (*p* > .05) (Figure 3A).

### RT-PCR detection of the relative TLR4, MyD88 and NF-κB mRNA expression levels

At 48 h after contrast agent injection, in comparison, the experimental control group showed significantly increased TLR4, Myd88, and NF-κB mRNA expression levels. After the action of the different atorvastatin doses, the various groups showed significantly reduced TLR4, Myd88, and NF-κB mRNA expression levels, which represented significant differences (*p* < .05) compared with the experiment control group. However, no significant difference was detected between the medium-dose and high-dose groups (*p* > .05) (Figure 3B).

### ELISA detection of the expression of the inflammatory cytokines IL-1β, IL-6, and MCP-1 in the centrifuged renal tissue supernatants from the various groups

**Figure 4.**
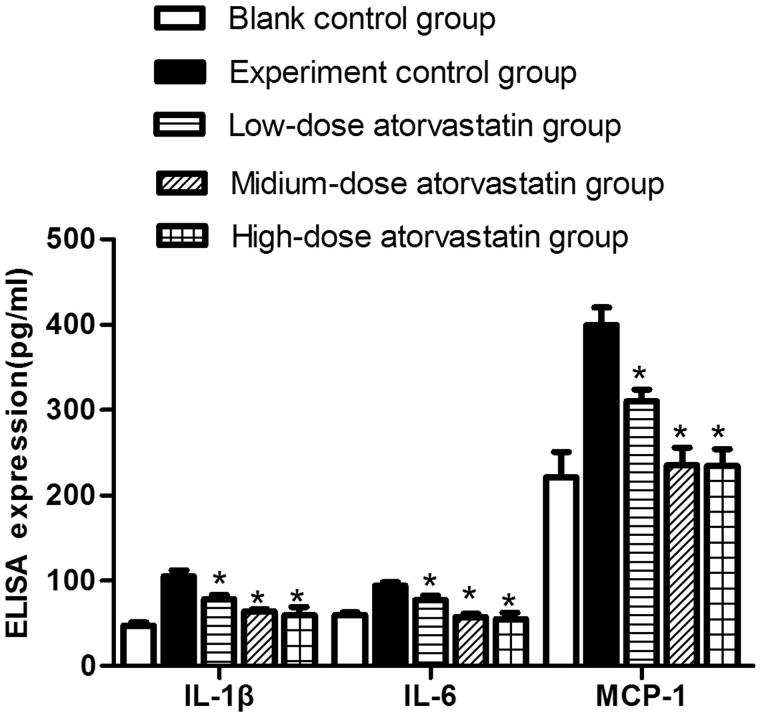
ELISA detection of IL-1β, IL-6, and MCP-1 expression in the centrifuged renal tissue supernatants from various groups * indicates *p* < .05 compared to the experimental group. All variables were evaluated 48 h after the induction of AKI.

At 48 h after contrast agent injection, ELISA was used to detect IL-1β, IL-6, and MCP-1 expression in the various groups. The experimental control group showed significantly increased IL-1β, IL-6, and MCP-1 expression compared with the blank control group. The different atorvastatin doses significantly reduced the expression of different inflammatory cytokines, including IL-1β, IL-6, and MCP-1; these differences were significant compared with the experiment control group (*p* < .05). However, no significant difference was detected between the expression levels in the medium-dose and high-dose groups (*p* > .05) (Figure 4).

## Discussion

The pathophysiology of CIN is complex. Inflammation probably followed an initial hypoxic and ROS-mediated injury, with a feed-forward loop of inflammation, vascular compromise and hypoxia; factors associated with inflammation include the oxidative stress reaction that releases oxygen free radicals, endothelial dysfunction or apoptosis, and renal vasoconstriction leading to renal medullary ischemia and hypoxia. The direct toxic effect of the contrast agent involves all aspects of cell metabolism, including dysfunctions in cellular energy metabolism, disruption of calcium homeostasis, changes in cell polarity, and apoptosis. A change in renal hemodynamics after the use of contrast agents is the initiating factor of CIN. Increase in renin, angiotensin, and other vasoconstrictor substances lead to the enhanced production of local adenosine, sodium ions, and endothelin, which cause renal vasoconstriction and renal blood flow redistribution, whereas the increase in vasoconstrictor substances is accompanied by the reduction of vasodilators (i.e. NO and prostaglandins), which lead to renal medullary ischemia and hypoxia [19,20]. The reduction in the renal medullary blood flow and oxygenation may induce renal lipid peroxidation and increase renal tubule transport activity, resulting in the production of oxygen free radicals (ROS) in the outer zone of the renal medulla. The generation of ROS further reduces the blood flow to the medulla and decreases the superoxide dismutase (SOD) and catalase (CAT) activity levels of the active oxygen scavenging system, causing renal tubular damage [21]. A pathological examination can reveal remarkable expansion of the tubular lumen, hyaline casts, flattened tubular epithelial cells, vacuolization, and focal interstitial inflammation.

TLR4 is an important member of the TLR family and is expressed in tubular epithelial cells, mesangial cells, podocytes, and other renal cells in addition to its wide distribution in neutrophils, monocytes, macrophages, dendritic cells, B lymphocytes, and other immune cells [22]. This receptor may be involved in the occurrence of renal interstitial inflammation and the repair of damaged renal tubules. TLR4 plays an important role in inflammatory signal transduction and is an important upstream immune response regulator that can activate immune and inflammatory responses by regulating the expression of its downstream signaling molecules, such as NF-κB. This process releases large amounts of inflammatory cytokines and effector cell molecules and eventually leads to kidney inflammation. TLR4 expression in the kidney is weak under normal circumstances. In contrast, the expression of some TLR4 molecules is increased in pathological states. TLR4 binding to the corresponding ligand triggers downstream signal transduction pathways, including Myd88 and the Myd88-independent pathway [23]. TLR2 and TLR4 are associated with renal ischemia-reperfusion injury (IRI) [22]. Stimulation of mouse renal tubular epithelial cells with lipopolysaccharide (LPS) can lead to increased TLR4 expression and chemokine molecule secretion [24]. C3H/HeJ mice with a TLR4 gene deletion do not show LPS-induced acute kidney injury (AKI), whereas mice with a normal TLR4 gene can present severe AKI. This phenomenon is thought to be related to the increase in TNF-α secretion. Recipient normal mice transplanted with TLR-4-deficient mouse kidneys do not show significant AkI. In contrast, recipient mice lacking the TLR4 gene transplanted with a mouse kidney containing a normal TLR4 gene present serious endotoxin-induced AKI [25].

Currently, statins are the basic medicine for anti-atherosclerosis treatment, have wide clinical applications, especially for cardiovascular diseases, and show high efficacies and good safety features. Given their pleiotropic effects, statins may exert effects on CIN pathogenesis via multiple routes, including improvement of blood flow dynamics, anti-inflammation, antioxidation, improvement of endothelial function, and anti-apoptosis. At present, many scholars believe that the anti-inflammatory mechanism of statins is related to the regulation of the downstream transcription factor NF-kB. NF-kB is the modulator of the expression of many inflammatory cytokines (i.e. IL-6, and TNF-a) [15]. Based on the modeling method of Goodman et al. [26], this study successfully established a rat model of CIN. After contrast agent was given, we observed that the level of kidney function in the rats in the experimental control group was significantly decreased compared to the blank control group. Moreover, high levels of kidney tissue infarction, casts, tubular epithelial cell swelling, transparentness, loss, hemorrhage, neutrophil infiltration, and the emergence of a large number of apoptotic cells were observed. After the actions of varying atorvastatin doses, the rats showed improved renal function, a reduced degree of renal tissue injury, decreased numbers of apoptotic cells, some decreases in TLR4, Myd88, and NF-κB protein and mRNA expression, and a significant decrease in the expression of NF-κB downstream inflammatory factors, such as IL-1β, IL-6, and MCP-1. These results show that the specific mechanism by which statins reduce CIN may be dependent on targeting TLR4, inhibiting TLR4 expression, and regulating the TLR4/Myd88 signaling pathway. Alternatively, statins may inhibit the expression of IL-1β, IL-6, MCP-1, and other downstream inflammatory factors to protect against renal tubular epithelial cell injury.

In summary, atorvastatin protected tubular epithelial cells from injury probably by suppressing TLR4 expression, regulating the TLR4/Myd88 signaling pathway, and inhibiting the expression of downstream inflammatory cytokines, such as IL-1β, IL-6, and MCP-1, indicating that TLR4/Myd88/NF-κB are indeed involved in the occurrence and development of CIN. However, because CIN is a complex pathophysiological process involving multiple factors, the consequent effects of this signaling pathway on the occurrence and development of CIN need to be investigated by more in-depth and meticulous studies.
